# Challenging the financial inclusion-decent work nexus: evidence from Cambodia’s over-indebted internal migrants

**DOI:** 10.1007/s43508-021-00026-7

**Published:** 2021-10-05

**Authors:** Nithya Natarajan, Katherine Brickell, Vincent Guermond, Sabina Lawreniuk, Laurie Parsons

**Affiliations:** 1 Department of International Development, King’s College, London, London, UK; 2 Department of Geography, Royal Holloway, University of London, London, UK; 3 School of Geography, University of Nottingham, Nottingham, UK

**Keywords:** Financial inclusion, Decent work, ILO, Debt, Microfinance, Cambodia, Migration

## Abstract

In this paper, we question the promotion of financial inclusion, and microfinance specifically, as a means to achieve ‘Decent Work’ (DW) under the International Labor Organization’s (ILO) programme. Drawing upon original research findings from two types of internal migrants in Cambodia, we make a twin contention: first, that excessive levels of microfinance borrowing by garment workers are part-outcome of the failings of the DW programme to engender ‘decent enough work’, and second, that microfinance borrowing is actually eroding rather than contributing to the prospect of decent work for debt-bonded brickmakers in the country. The data presented on two of the largest sectors contributing to Cambodia’s growth in recent decades, enable the paper to show how microfinance and labour precarity are intertwined through the over-indebtedness of workers in both cases. The paper ultimately looks to caution the ILO on its current promotion of financial inclusion and microfinance in particular, stressing the need for significant sectoral reforms before this form of credit can be considered to align with the core principles of the DW programme.

## Introduction

‘The ILO has found microfinance to be an invaluable tool within its programmes in helping to reduce poverty and eliminate child labour and debt bondage. The goal is financial security, a key aspect of decent work.’ - ILO (2005)

‘Decent work sums up the aspirations of people in their working lives. It involves opportunities for work that is productive and delivers a fair income, security in the workplace and social protection for families, better prospects for personal development and social integration, freedom for people to express their concerns, organize and participate in the decisions that affect their lives….’ - ILO (2021)

In 2019, microfinance—the ‘new subprime frontier of millennial capitalism’ (Roy, 2010: 218)—reached an estimated 140 million people with loans totalling an estimated US$124 billion (Convergences, 2019). Microfinance mobilises narratives that portray finance as empowering and liberating (Mader, 2015; see also Schwittay, 2014). However, a shift in the mission of microfinance to alleviate poverty has taken place, from state-backed microloans for entrepreneurship, to a broader, commercial agenda of financial inclusion (Mader & Sabrow, 2019). Often portrayed as the only legitimate source of ‘pro-poor’ financial products and innovations, private players now provide not only lending but also savings, insurance and, importantly, all kinds of payment products and services (Mader, 2016). However, as Mader (2018: 463) remarks, ‘much of today’s financial inclusion activity is still microfinance: small, short-term, high-interest loans extended to low-income people’.

In this paper, we question the promotion of financial inclusion, and microfinance specifically, as a means to achieve ‘Decent Work’ (herein DW) under the International Labor Organization’s (ILO) programme. The ILO launched the DW programme in 1999 under the aegis of its first global South Director-General Juan Somavia. It was seen as a culmination of efforts by the ILO to try and distance itself from the proliferating impacts of neoliberal globalization on declining labour standards (Vosko, 2002), and to move beyond its historically capital-centric stance (see Cox, 1977), though critics have pointed to its lack of legal mandate and concurrent enforceability (Lerche, 2012), and the dischord between rhetoric and reality in DW policies (Hauf, 2015). It is this theme of discord which this paper elaborates on and evidences, problematising the ILO’s position (cited above) that microfinance is an ‘invaluable tool’ in achieving DW and financial security for workers and their families. Drawing upon research findings from two types of internal migrants in Cambodia, we make a twin contention: first, that excessive levels of microfinance borrowing by garment workers are partly the outcome of the failings of the DW programme to engender ‘decent enough work’, and second, that microfinance borrowing often erodes, rather than contributes to, the prospect of decent work for debt-bonded brickmakers in the country.^1^

While the view that financial inclusion is beneficial to building DW has been espoused for several decades, the links between financial inclusion and the ILO also predate the DW programme. Their longevity underscores the difficult task of challenging this financial inclusion-decent work orthodoxy. The ILO was a founding member of the Consultative Group to Assist the Poor (CGAP), a global thinktank which drives forward the financial inclusion agenda across the development sector, and engaged in small-scale credit programming as far back as the early 1990s (Bernards, 2016). In 1998, just prior to the launching of the DW programme, an ILO report (see Balkenhol, 1998) identified the potential of microfinance programmes in tackling unemployment and assisting marginalized populations through promoting enterprise creation. As Gravesteijn (2014) has argued, once the DW programme was launched, there were numerous links made between DW and paths out of poverty via microfinance, including helping the poor to manage risks, employment generation, bettering working conditions, and women’s empowerment.

In 2014, a comprehensive report entitled ‘Microfinance for Decent Work: Enhancing the Impact of Microfinance’ brought together this agenda (see ILO, 2015). The report presented findings from an ILO collaboration with 16 microfinance institutions from 2008 to 2012. This action research programme looked to how microfinance programmes could address a range of issues related to DW, including eliminating child labour, formalizing enterprises, and reducing vulnerability. The report’s findings were mixed, and did not reference the wealth of critical literature, detailed in this paper and our findings, which point to issues in the proliferating financial inclusion agenda. Nevertheless in 2015, the newly launched Sustainable Development Goals included one goal (8) explicitly targeted towards the DW agenda, entitled ‘Decent Work and Economic Growth’. It includes a target (8.3) advocating ‘access to financial services’ as a means of supporting ‘productive activities, decent job creation, entrepreneurship… [and] the formalization and growth of micro-, small- and medium-sized enterprises’ (UN, 2015).

On the basis of our empirical data on migrant garment and brickmakers, we challenge what we are calling the ‘financial inclusion-decent work nexus’, which remains powerful in the ILO and in parts of the broader international development arena. The original data presented from the garment and construction sectors, two of the largest sectors contributing to Cambodia’s growth in recent decades, enable the paper to show how microfinance and labour precarity are intertwined through the over-indebtedness of workers in both cases. Over-indebtedness here refers to when a borrower ‘is continuously struggling to meet repayment deadlines and structurally has to make unduly high sacrifices related to his/her/[their] loan obligations’ (Schicks, 2013: 1239).

While the ‘Better Factories Cambodia’ programme represents a flagship ILO intervention in Cambodia’s garment industry, we demonstrate how efforts to foster DW (including contracts and social security for workers) have not precluded the need for some workers to take on significant loans to supplement low wages and make detrimental sacrifices to repay them. These are examples of debts which occurred after having arrived in-destination, ones which are rarely examined in comparison to those motivating migration in the first place (Bylander, 2020). Yet some garment workers are also embroiled in repaying microfinance loans taken on by rural family members back home, which they must remit to, and which can drain their already-meagre incomes. Cambodian brickmakers’ experiences of adverse consequences from financial inclusion via microfinance echo those of garment workers in Cambodia’s regulated factories. In this latter case, we explore how rural precarity can be exacerbated through the taking on of microfinance loans, linking the particular conditions of loans to why many people decide to move to debt-bonded labour in unsafe conditions on brick kilns.

Today, Cambodia represents one of the ten fastest-growing economies in the world over the past 20 years (ILO, 2019a). The ‘miracle’ (World Bank, 2018) of its post-conflict transition is crowned by graduation to lower-middle-income status in 2015, forged through a structural transformation from a primarily rural and agriculture-based economy to an increasingly urban, industrial and services-based one (Diepart et al, 2019). Yet crucially, in keeping with many countries across the region, transition and the ensuing rural dispossession that has taken place have been met by low levels of labour absorption into formal sectors of work (Diepart et al, 2019). With this, since the late 1990s and early 2000s, labour migration has rapidly accelerated. This began with intra-provincial and very short-range migrations but has grown to encompass longer-range internal movements to key urban zones where the garment and construction sector work has seen significant expansions.

Although migration has been shown to be critical to the management of debt within poorer households, with (over)-indebtedness sometimes motivating, if not compelling, people to migrate in the first place (e.g., Mosse et al., 2002), what has remained relatively unexplored are the increasing links between a particular type of debt—microfinance debt—and migration and mobility. The shared exposure of garment and brickmakers to migratory pressures and labour precarity that stems in part from over-indebtedness to microfinance lenders, works to demonstrate how financial inclusion and decent work are not necessarily coterminous. The relationship between the two is more contradictory than current ILO policy positions and interventions suggest.

To challenge the ‘financial inclusion-decent work nexus’ then, in the next section we scrutinize claims made through a review of critical literature from labour studies, economic geography and political economy. We then provide key information and context to the studies and methods used in developing our argument. The empirical heart of the paper is presented across two case studies of ‘not decent enough’ and ‘indecent’ work in the garment and brick industries respectively. Lastly our conclusion returns to, and reiterates, the ambivalence of the links which exist between financial inclusion and ‘decent work’.

## The financial inclusion-decent work nexus: a challenge

After several decades of expansion, the capacity of microfinance to ‘make poverty history’ has been called into question. There is a growing consensus that microfinance has not been effective at increasing household incomes, alleviating poverty and facilitating broader development outcomes (Kar, 2018; Mader, 2018). Duvendack and Mader (2019: 7), in their systematic review, found that ‘the effects of financial services on core economic poverty indicators such as incomes, assets or spending, and on health status and other social outcomes, are small and inconsistent’. Furthermore, far from the promised beneficial outcomes, microfinance has often been called into question for increasing the reliance of marginalized and low-income workers, as well as unemployed or underemployed people, on credit for social reproduction (e.g., Soederberg, 2014; Taylor, 2012), reproducing inequalities and, specifically, unequal gender relations (e.g Kar, 2018; Roberts, 2015; Young, 2010), as well as causing extreme stress to its users (e.g., Rankin, 2013). This paper contributes to this critical literature on microfinance and further problematises the links between this development project and labour conditions.

Broadly speaking, the ideological basis for using credit, most commonly microfinance, for achieving the ends of the DW programme is centred on the idea of market integration, where poverty is conceptualized as representing exclusion from so-called ‘formal’ growth and markets:

‘Microfinance is the provision of financial services to the poor on a sustainable basis. It means credit for income generation, for starting or expanding micro-enterprises; it also means savings, emergency loans, remittances, guarantees, payment services and insurance. Microfinance helps the working poor—employed or self-employed—in a variety of ways: to raise and diversify incomes, to package incomes around wage earnings, to manage risks, stabilize incomes and reduce vulnerability’. - ILO (2007)

The underlying assumptions made here suffer on two fronts: the financial inclusion-decent work nexus fails to acknowledge (1) how integration into markets, be they labour markets or credit markets, on *adverse* terms risks deepening poverty rather than alleviating it (Hickey & du Toit, 2007); and (2) how microfinance and entrepreneurship are not necessarily co-joined.

Taking these in turn, microfinance loans, specifically, have become considerably more adverse for debtors, as the sector as a whole has become unmoored from the constraining forces of state regulation, and transformed into an increasingly commercial sector (Bateman, 2010). This is due to a concerted shift from the developmental aims of early microcredit programmes, largely centred on providing low-risk, state-backed loans to women with savings groups acting as collateral, to the current, more individualized model which is dominated by private enterprises and seeks out new debtors, leading to increasingly risky loan packages for increasingly precarious populations (see Roy, 2010; Ghosh, 2013). In the late 1990s in Cambodia for example, international donors and agencies supported the launch of several microfinance institutions, aiming to replicate the microcredit model pioneered by the Grameen Bank in Bangladesh in the 1980s. ACLEDA, the first institution to provide credit to small groups of women in rural areas, started its operations as a non-profit NGO in 1991, supported by the ILO amongst others (Green, 2020a). From the early 2000s, experts in the development community strongly advocated for the transformation of microfinance institutions into for-profit commercial enterprises, echoing a broader shift towards neoliberal market-based approaches to development in Cambodia, and beyond (Green, 2020a).

The commercialisation of microfinance institutions in Cambodia has seen the sector expand at an extraordinary rate in the past 20 years. The Cambodian microfinance sector is heterogeneous, comprising both formal and semi-formal institutions of varying sizes, offering different loan conditions and requirements for borrowers. Between 2000 and 2019, the number of borrowers increased from about 175,000–2.6 million people (CATU et al., 2020). With Cambodian borrowers holding more than US$10 billion in microloans in 2019, Cambodia constitutes one of the largest microfinance industries in the world in terms of borrowers per capita (CATU et al., 2020). Furthermore, the average loan size in Cambodia—US$3804—constitutes by far the highest average loan size in the world and far surpasses GNI per capita in the country (CATU et al., 2020: 1). This expansion in the number of borrowers and loan volumes, in tandem with relatively weak non-binding regulations, has contributed to large profits in the sector (Bateman, 2017). The Cambodian Microfinance Association (CMA), comprised of senior managers of large commercial banks (Bateman, 2020) promotes continued self-regulation of the industry (Green, 2020b), describing itself both as ‘an NGO and professional association that aims to ensure the prosperity and sustainability of the microfinance sector in Cambodia’, and its activities as having enabled ‘each member microfinance institution to become stronger and more successful and thereby attract support from the international market to enable industry expansion’ (2021). Leading lenders in the country however have worked to circumvent interest rate caps introduced in March 2017 at the behest of the Cambodian government ‘by quietly imposing a new range of fees and charges on clients’ (Bateman, 2020: 4). As Heng et al., (2021: 15) comment, ‘issues of over-indebtedness and irresponsible borrowing and lending, and a loss of lending discipline’ were ‘at the heart of the concerns which triggered the introduction of interest rate cap by the government’. Continued actions to circumvent the impact of the interest rate cap reflect the continued emphasis on maintaining growth and profit, and sit in tension with the CMA’s promotion of Smart Certification (a scheme which ran from 2013 to 2020) for its MFI members in which client protections and responsible lending are core tenets (Green, 2020b).

Commercialisation has had profound impacts both beyond Cambodian borders and in the country. On the former front, profits for shareholders—nearly 90% of whom are based outside Cambodia (Sinha, 2014)—of the seven largest microfinance institutions are among the highest in the global South (Mahanty and Green, 2020). Cambodia now accounts for 8.4% of all microfinance-specific investment in the world and is ‘second only to India, which has a population nearly 85 times greater than Cambodia’ (Green, 2020a: 5). On the latter front, both independent and industry-funded studies have demonstrated increasing problems of over-indebtedness, with between 28 and 50% of microfinance institutions’ borrowers being either over-indebted or at risk of over-indebtedness (CATU et al., 2020; Liv, 2013; Mahanty and Green, 2020). As a result, borrowers juggle a wide range of intertwined coping mechanisms, including, lower food quality and intake (Bateman, 2017); distress land sales (Green, 2019; LICADHO, 2019), cross-borrowing (Liv, 2013) as well as cross-border migration (Bylander, 2014, 2019a, 2019b; CATU et al., 2020); to continue repaying loans. In relation to cross-border migration, microfinance debt-driven migration has been a prevalent phenomenon for years not only in Cambodia (Bylander & Hamilton, 2015; Green & Estes, 2019; Ovesen & Trankell, 2014) but also in countries such as Guatemala (Heidbrink, 2019; Johnson & Woodhouse, 2019; Stoll, 2012). For instance, Johnson and Woodhouse (2019) show how microfinance loans are used by migrants to pay coyotes (guides) to facilitate border crossing to the US. In Cambodia, over-indebted people migrate to countries such as Thailand and Malaysia in search of work to repay microfinance loans (Bylander & Hamilton, 2015; LICADHO, 2019). As a recent report by LICADHO uncovered (CATU et al., 2020: 1), microfinance institutions in some instances encourage migration ‘by withholding loans unless a family could prove they had at least one member working abroad’. Scholars and civil society organisations have warned that debt-driven, or distress, international migration is a particularly precarious form of migration that puts migrants at greater risk of abuse and exploitation as they cross borders (LICADHO, 2019, CATU et al., 2020). In this paper, however, we suggest that internal migrants also contend with significant risks linked to their over-indebtedness and labour precarity; which warrant greater attention. After all, Cambodian migration remains, by and large, concentrated within its national borders.

Second, and relatedly, the financial inclusion-decent work nexus fails to acknowledge how microfinance and entrepreneurship are not always co-joined. As Bateman (2010) has argued, the assumption that there is limitless space for micro-enterprises in village economies in particular fails to grasp the limits of such a model, where local purchasing power and low population size severely restrict the potential for market competition among multiple small enterprises. As such, a wider literature has highlighted how microfinance loans are largely used for daily reproductive needs (Guérin, 2014), in a broader context of chronic un- or under-employment, low wages, and scant social protections across the Majority World (Breman & van der Linden, 2014). This is shown to be the case in Cambodia, where ‘household debt has soared’ and microfinance loans are used to ‘cover basic life needs within the family, such as food, healthcare, and education’ (Green & Estes, 2019: 130; see also Liv, 2013).

## Researching the lives of Cambodia’s overindebted internal migrants

Analysis in this article is drawn from across multiple research projects, spanning four years (2017–2021), split across the two case studies. First, the garment sector case study draws upon data collected from two research projects. The earliest (2017–2020) examined trade unions in Cambodia’s garment sector, and the gendered norms and experiences of labour organising within this. The researcher conducted a series of observations and more than 100 interviews with garment workers, trade unionists, labour rights advocates. The qualitative interview data presented in this paper is drawn from this project. The quantitative survey data in our empirical analysis is taken from the latest project (2020–2022), exploring the economic impacts of COVID-19 on female garment workers and the existing and newly emerging social protections made available to assist them. This ‘ReFashion’ project employs a mixed-method approach, combining a quantitative survey of 203 female workers and follow-up qualitative interviews with 60 workers from the original sample (longitudinal work is ongoing). The survey recorded, with the use of software, women’s employment changes and the financial, health, and wellbeing impacts on both workers’ immediate household and on rural families. The first phase of the study began in October 2020, with quantitative data collection completed between November and December 2020. The survey captures the indirect economic impacts of the pandemic, given that at this point Cambodia recorded zero deaths and less than 500 COVID-19 cases. In this first round of research, conducted mainly in-person given the very low numbers of COVID-19 cases at the time, workers were recruited with the assistance of labour unions and the Cambodian Ministry of Labor.

Second, the brick sector case study is drawn from the ‘Blood Bricks’ research project exploring the links between climate and debt bondage in the Cambodian brick-making sector (Brickell et al. 2018; Natarajan et al. 2021). Data collection for analysis in this paper comprised two key aspects. First, 81 interviews were conducted with brick workers, brick kiln owners, former brick workers, and various other relevant authority figures, in 30 kilns located in and around Phnom Penh, from 2017 to 2018. Interviews explored levels, type and structure of debt, reasons for debt-taking, working and living conditions on kilns and aspirations among workers. In 2018, a quantitative survey was conducted in three villages with high levels of out-migration to brick kilns. The total sample of 308 households comprised all brick kiln-sending households and a randomized sample of non-brick households across the three villages. The survey covered demographic data, asset ownership, farming and non-farming production and labour activity, debt type, level and structure, and wider livelihood strategies. This was supplemented with in-depth qualitative interviews with selected villagers, including people from brick kiln and non-brick kiln-sending households, local authority figures, and religious figures, to better-develop an understanding of the debt context.

## ‘Not-decent-enough work’: microfinance-dependency and sacrifice in the garment sector

Since its inception in the mid-1990s, Cambodia’s garment sector in particular has driven Cambodia’s economic dynamism, growing from a US$27 million industry in 1995 to exporting over US$8 billion in 2018 (ILO, 2019a). The sector accounts for 75% percent of merchandise exports and is estimated to contribute to 20 percent of total Cambodia’s gross domestic product (GDP). As the garment industry has grown to dominate Cambodia’s economy, it has swallowed an increasing share of the country’s labour reserves too. A workforce of 18,000 in 1995 (Bargawi, 2005: 9) has swelled to 750,000, some 6% of the total labour force (ILO, 2019a). With a significant informal workforce in other sectors across Cambodia as stated above, garment workers are often assumed to be among the lucky ones: key beneficiaries, as well as drivers, of Cambodia’s economic boom. Compared to the dehumanising and physically degrading labour of the brick kilns, the garment sector offers salaried work, limited social protection provision through a contributory health care and employment injury scheme, and a minimum wage—with the industry still the only sector in the country covered by Cambodia’s minimum wage law. The garment sector represents something of a flagship programme for the ILO’s DW agenda, through the ‘Better Factories Cambodia’ scheme which oversees the sector and is responsible for many of the gains in terms of social security.

Stubborn ‘decent work deficits’ that are also evident across the garment industry globally remain, however. Where trade liberalisation has fostered increasing international competition in an already densely crowded garments market, downward pressure on unit prices has been exacted on factory profits, with deficits paid from workers’ pockets. Flexibilisation and intensification of work patterns have resulted, with employers resorting to increasing use of 3- and 6-month fixed duration contracts and escalating production targets to extract ever greater margins from an increasingly exploited workforce (Human Rights Watch 2015). Reflecting the spectre of what we have labelled not-decent-enough work, between 2001 and 2011, as GDP growth and consumer inflation soared, the real wages in the sector fell by 22%, locking wages at levels below basic reproduction requirements (Selwyn, 2019).

The COVID-19 crisis has brought to the fore the arguably insecure nature of the economy, dependent on uneven and unreliable integration into circuits of global trade. In the year since March 2020, some 150,000 of the garment industry’s workforce have been suspended or laid off, as the effects of successive manufacturing and consumer lockdowns ripple through global supply chains (Arnold, 2021). This unprecedented unemployment event has highlighted the precarious nature of work in the garment sector prior to the pandemic, where personal debts have been taken on to subsidise sub-subsistence wages. Tallying with figures from Cambodian non-governmental organisations (cf. CATU et al. 2020; LICADHO, 2019), the ‘ReFashion’ survey of 203 female garment workers affected by COVID-19 production disruption found that 123 women—or 63%—reported outstanding loans borrowed before the pandemic.

The most common sources of borrowing for garment workers were formal organisations like banks and microfinance agencies (44% and 26% of individual loans, respectively, *n* = 162), followed by informal sources such as family or friends (20%), and moneylenders (7%). The formal agencies were responsible for lending the largest sums: a mean average loan of $7000 from the bank and $5077 from an MFI, contrasted with $1455 from family or friends, and $1134 from a moneylender. Far beyond the characteristic of “micro”-credit, this formal lending elevated the average loan portfolio of women in the sample to $4,731, more than double the basic yearly salary of a worker in the sector, fixed at $192/month. The ability to take on debt levels that are incongruous with earnings speaks to the deregulation of microfinance loans discussed earlier. Furthermore, corroborating suggestions that remuneration falls under living wage levels, very few of these individual loans were described as facilitating entrepreneurial potential through agricultural (2%, *n* = 162) or trade investments (5%), for example. Instead, they were typically used alongside income to support everyday household expenditures (Bylander, 2014; Bylander & Hamilton, 2015). After investments in land and housing (31%); 23% of loans were taken out to purchase transport, including motorbikes; 22% were to support daily living like food and rent; and 16% to cover health or medical care. These figures suggest that garment work in Cambodia has not become ‘decent enough’ to preclude workers’ reliance on credit to meet the costs of everyday social reproduction.

As such, trade unions argue that the garment sector’s contribution to poverty reduction in Cambodia may be superficial. “For the gift of microfinance, the worker’s living condition is better but … every one of them is in debt”,^2^ one national union leader commented. Another agreed, “We might think that the workers are better off now because they have more salary. Some workers have a motorbike and money to build a house. [But] in 1990, there were not many banks and the availability of loans was limited. Now they can borrow money from the bank to buy materials. Therefore, workers are seen to be better. They have debt with the bank. Every month they work only for paying back to the bank”^3^ (General Secretary, trade union federation, February 19th 2020). These suggestions build on critical work that challenges the purported benefits of value chain integration for workers as a ‘mirage of development’ (Crossa & Ebner, 2020: 1218), masking conditions of exploitation that may also prosper in labour-intensive and low value-added segments of production chains, like the Cambodian garment sector.

The most frequent source of collateral for debt in the ‘ReFashion’ study was residential land, constituting 41% (*n* = 162), against 38% of loans that were unsecured and a further 20% borrowed against agricultural holdings. As stated above, the consequences of any default or inability to make repayments can be severe, particularly as 3- and 6- month fixed-term contracts are becoming an industry norm. “Many [workers], before, they worried a lot about their stomach. They don’t know if they will have money to buy vegetables or rice to eat”,^4^ a trade union leader confirmed. “But now, when I ask them, the first mouth opening says ‘microfinance’… ‘*Knyom peak luy te!* [I owe money!] I owe money to the bank. *Knyom peak luy te!* [I owe money!] How can I get money to pay my debt?’”. An investigation by a local civil society organisation found that 96% of garments workers with loans said their life was either “much worse” (80%) or “slightly worse” (16%) now, compared to before taking out the loan (CATU et al., 2020: 1). Our own data indicates that many workers face considerable difficulties in managing existing debt burdens. A further 16% of loans taken to prior to the pandemic, for example, were described as additional credit borrowed to make loan repayments where existing debts had already become unsustainable. The financial inclusion-DW nexus is thus not guaranteed. Rather, the sheer scale of microfinance-dependency amongst Cambodian garment workers prior to, and during, the Covid-19 pandemic reflects the ‘not-decent-enough’ character of work in the sector.

Evidencing this further, we found that many women support multiple debt burdens through their garment income. Indeed, Cambodian households rely heavily on labour wages to service loans (Green & Estes, 2019). With work and employment opportunities limited in rural areas, many workers migrate to the city to find waged income, continuing to provide financial support to rural-based spouses, parents or other family members linked by translocal, filial webs of mutual dependency and obligation (Lawreniuk & Parsons 2020). The 203 female workers in the ‘ReFashion’ study, for example, provide monetary support to a further 142 households through regular remittance payments, with monthly receipts averaging $77 before COVID-19. After daily expenditures (85%, *n* = 142), health (41%), and childcare (32%), loan repayments (27%) were the fourth most commonly reported use of income. Trade unions suggested that the true figure could be much higher. “If we ask the workers, they don’t know because their parents are the people who borrow the money”,^5^ one leader explained. “But the children work in the factory to pay back the debt.” The channelling of remittances into formal financial circuits through, for instance, the repayment of microfinance loans, has recently been located within broader processes of remittance marketisation and financialisation (Guermond, 2020; Datta, 2017; Kunz et al, 2020). These analyses have foregrounded the disciplinary role of a migration-development agenda which can push ‘migrant workers and their families to dedicate an ever-increasing fraction of their wage remittances to the management of formal financial debts and assets to secure their future’ (Datta and Guermond 2020: 331).

This state of indebtedness depicted by the union leader was not only viewed as a product of precarious conditions in the industry, however, but also an indirect driver. As trade unions explained, the financial pressure of repayment compels workers to accept unfair and discriminatory practices in the sector, limiting their capacity to engage in collective action to resist the worsening terms of their employment. “For this point, we are aware of it clearly”,^6^ emphasised the same leader. “For some workers, they have a willingness to join with the union because they don’t have any debt. They protest, strike and demonstrate. They can do this because their family do not have any debt. Other workers, they don’t dare because their family have debts.” Another described workers’ inability to resist the intensification of work patterns in the sector, providing an example of a factory that had increased workers’ production targets from 500 to 600 units—an increase of 20%, apparently to compensate for the expected impacts of tariffs imposed by the EU. “Even though they increase productivity, the workers will still work for the factory, I think”,^7^ he surmised. “They don’t have an option. [It’s] like what I said before. There are very few workers who do not have debt with the bank. Because most of them have debt, they have to work.”

Here, and in contrast to how decent work is defined by the ILO in the direct quote at the beginning of this article, over-indebtedness—resulting from the expansion of credit provision combined with low wages—leads to insecurity in the workplace and constitutes a significant barrier for workers willing to organize, unionize and express disagreements related to working conditions. What this also shows is that an understanding of the financial inclusion-decent work nexus requires paying attention to the increasing role of mobility in supporting and defining livelihoods within households and villages. As Lawreniuk (2016) writes, labour migration in Cambodia is not unilinear, but rather complex and churning, as migrants abandon neither the countryside, nor their engagement with agriculture, but utilise the modern sector to enhance their rural livelihoods via bi-directional remittance, which flows throughout Cambodia in vast sums. Ultimately then, financial inclusion risks exacerbating poor working conditions for Cambodian garment workers and precluding them from engaging in labour organising. These findings speak to the need for greater caution in linking DW with financial inclusion.

### Indecent work: microfinance borrowing and debt bondage of brickmakers

Of the numerous migrant occupations undertaken by Cambodians both within and beyond the country’s borders, brick work is often seen as being amongst the least desirable. In the words of Kosal, a debt-bonded brick worker living and labouring in the outskirts of Phnom Penh, ‘We have to work here because of debt; if not we would have decided to leave this place long ago. This is our unsolved problem’.^8^ In this case study of brickmakers, we show how despite ILO mantra financial inclusion for brick workers has not led to financial security, a core dimension of the decent work agenda.

How then does financial inclusion lead to debt bondage on the kiln? Interviews with kiln workers revealed that the key driver for taking on a debt bond and becoming tied to brick kilns was unsustainable levels of microfinance debt. Surveys across three sender villages revealed that an average of 54% of households were indebted, with microfinance debt constituting 93% of all loans taken in surveyed households. Crucially, different levels of debt speak to different levels of desperation vis-à-vis the labour market, where brick kiln-sending households held average debts of US $1380.93, compared to an average of US $814.72 among non-kiln-sending households. Thus households with greater debt levels tended to be more desperate for work, compelling them into more indecent parts of the labour market, and into debt bonded labour arrangements which offer immediate capital to repay existing loans. Furthermore, despite the heterogeneity of the lending market, our data revealed that borrowers from both brick and non-brick households owed their largest loan to a relatively small pool of large, formal lenders. 80% of brick and non-brick workers respectively indicated they had taken their single largest loan from one of the top ten microfinance institutions^9^ in the country.

We suggest here that particular conditions of microfinance loans from these larger players contribute to existing precarity among such households, leading then to debt bondage. We also acknowledge a variety of related issues encountered in the field which contribute to over-indebtedness, including taking on multiple loans from both formal and informal lenders, and the practice of ‘extend and pretend’ (Bateman et al., 2019)—where lenders continue to issue new loans to debtors before old ones are repaid. We contend that these issues stem fundamentally from the deregulation and commercialisation of the microfinance sector.

Looking first to the existing precarity of debtors, rural Cambodians have faced a reproduction squeeze in recent decades. Despite land redistribution in the post-Khmer Rouge era, the system of collective farming implemented by the People’s Republic of Kamupuchea (PRK) government in the 1980s proved unsuccessful, with many choosing to sell their small parcels of land as even subsistence cultivation proved difficult in a context of scant state support (Frings, 1994). Amongst those households with one or more member working on brick kilns, only 23% reported owning commercial farmland. The remaining 77%, therefore, reproduced themselves through a combination of insecure agricultural and non-farm waged work, and subsistence cultivation. Among the 23% that did own farmland, interviews with brick workers suggested that it was often efforts to improve agricultural productivity which led farmers to debt bondage on brick kilns. As male brick worker Sangha reported, ‘it was very difficult to grow the rice in the dry season because we didn’t have enough water… we needed to use a lot of capital, so we borrowed the money to buy oils, fertilizer, and it was difficult to pay it back…I sold my land to pay them [MFI institutions] off’.^10^ The state’s support for agricultural productivity-raising has been lacking since neoliberal restructuring in the 1990s (ADB, 2014), leaving farmers to contend with rising prices for agricultural inputs, irrigation, and an unpredictable, unregulated marketing system in which to sell their crops. Furthermore, Cambodia’s changing climate has exacerbated rural precarity (Natarajan et al., 2019), with an increasingly concentrated and erratic rainy season making crop failures more likely due to flooding, drought and pests (Thoeun, 2015).

Aside from agricultural investments and daily costs for social reproduction, the survey and interviews revealed that another key driver for debt-taking was medical expenses, for health issues incurred both on and off the kiln. As Phhoung tells us, ‘My son became sick, so we mortgaged our farm lands to get a loan. Then both my son and his wife went to work in a brick kiln and left their children with me so they could continue with their studies’.^11^ Cambodia’s poor health and social protection coverage is well documented, sometimes leading to out-of-pocket health expenses that risk tipping households into distress (Dalal et al., 2017), or in this case, debt bondage.

Taken together, rural distress and poor health coverage constitute a precarious context in which loan-taking provides a key lifeline for many. Yet the particular nature of microfinance loans and how they are structured are shown to compel these precarious households into debt bondage. As highlighted earlier, the microfinance sector in Cambodia has become increasingly commercial, with newly established motives for profit-seeking driving a deregulation of loan amount, collateral requirements, and loan requirements on the ground (Bateman et al., 2019).^12^ This means that households with poor income-generating prospects are able to take on multiple loans, or loans that far exceed their income capacity. The rigidity of repayment schedules jar with the insecure and temporally diffuse nature of available work, hence leading to what Saiag (2020, 23) terms ‘the mismatch between the time of labor and the time of finance’. Furthermore, acceptable forms of collateral have also been deregulated. As Green (2019) has highlighted, the business of valuing land for microfinance loans is a constructive one, reliant on numerous and often subjective factors to translate land into value. In our study villages, only 37% of those that had taken on forms of credit possessed any agricultural land. For the remaining 73%, the land title for the land on which their house sat—their homestead land—acted as a highly risky form of collateral. As Phala stated, ‘if we use the house as collateral and we do not pay back our loan to ACLEDA, ACLEDA will take our house and sell it at auction at minimum price’.^13^ The common phenomenon of using homestead land—a significant tie to one’s natal village as well as a key basis for social reproduction for women day-to-day—as collateral thus disciplines debtors who are at risk of losing access to such land.

The brick sector has been a willing recipient of bonded workers. As Cambodia’s supply of precarious labour increases as a result of the aforementioned factors, domestic demand for bricks has risen rapidly, due to a substantial boom in urban construction. As of 2019, the country contained some 464 operational kilns, with an average age of just over 15 years, reflecting the growth of Cambodia’s booming urban infrastructure. As of 2020, the population of the brick industry—i.e., those residing within brick kilns—stood at 10,217 people, of whom 4777 were female, 5440 were male and 3937 were aged under 18 (Author D and Co-author, 2020). The high number of children reflects an endemic problem of child labour long-noted of the industry (Authors, 2018; LICADHO, 2017). Equally, however, it reflects an industry characterised by its residential nature. The majority of brick workers live and work on kiln sites, residing in low-quality housing, usually situated a few metres from the kilns themselves. Commonly, only one member of each family is formally paid for the materials that they produce, the rest being shared by the family after payment. Overall, labour’s share of the final sale price of bricks constitutes just 8% (Authors, 2018).

Viewed thus, the economic incentive for workers to enter brick work appears limited. Yet it is not wages in themselves that induce workers to enter brick work, rather it is the availability of a debt bond, and the lack of wider options given the urgency felt by highly indebted households to repay microfinance loans. Brick kiln owners offer to repay prospective workers’ existing debts, on the basis that they work for the brick kiln owner until the newly transferred debt is repaid. Nevertheless, this is more difficult in practice than it often appears to those who enter the industry, as regular rain-induced stoppages, low wages and ill health see few able to repay much of what they owe (Authors, 2018). Consequently, brick workers may spend years or even decades in the brick industry. Furthermore, though workers may endure it for long periods, brick work is a dangerous and physically degrading occupation. Serious injuries, exhaustion and under-nutrition are endemic in the industry (Authors, 2018; LICADHO, 2017), whilst carrying bricks in a climate which averages almost 35 degrees during parts of the year is itself a substantial danger to health (ILO, 2019c). Combined with hours spent stoking kilns which can reach up to 1100 degrees (GKSPL, 2016), brick work reaches ‘the upper limits of what humans can tolerate before risking serious impairment’ (Lundgren-Kownacki et al., 2018: 347).

Despite very little economic incentive to remain working in the brick industry—and significant physical incentives not to—this is an industry held together on the basis of debt bondage, incurred through unsustainable microfinance debt-taking outside the kiln and wider structural precarity. The particular loosening of microfinance loan restrictions places rural indebted workers and farmers in a structurally insecure and often desperate position vis-à-vis the labour market, driving them to accept highly exploitative labour conditions and even spatial and temporal bondage in exchange for a release from the unceasing rhythms of microfinance repayments. ILO (2005) claims that microfinance is significant in the fight against child labour and debt bondage are therefore tempered as seen via the experiences of over-indebted brick workers in Cambodia where excessive microfinance borrowing can combine with structural precarities to drive some adults and children to work in kilns as work of the last resort.

## Conclusion

In this paper, we have looked to challenge the financial inclusion-decent work nexus. Drawing upon the experiences of Cambodian domestic migrant workers in two different sectors, our argument is two-fold. First, we contend that attempts to champion DW in the garment industry have not necessarily succeeded in preventing migrant workers from having to rely on microfinance debts to support not only themselves in destination areas, but also often-indebted rural family members back home. As wage levels remain below basic reproduction requirements, workers are shown to be pushed into debt and in some cases, compelled to accept hyper-exploitative, intensifying and discriminatory working conditions and practices. Having debts to repay, they also find themselves unable, or at least more hesitant, to unionize and organise on the shop floor. Excessive levels of indebtedness are both partly the result of, and conducive to, what we have labelled not-decent-enough work in the garment sector. Second, we argue that microfinance borrowing can directly exacerbate rural precarity and lead to debt bondage on brick kilns. Here, the particular nature and structure of microfinance loans (e.g., collateral requirements, rigidity of repayment schedules and deregulation of loan amounts) act as a form of compulsion into indecent work for debt-bonded brickmakers in the country, where the access to debt bonds provide relief from unceasing microfinance repayment schedules. In the two case studies, compounding structural precarities combined with microfinance borrowing result in types of work that do not fulfill the promises of the ILO’s DW programme.

Building on our insights then, we look to caution the ILO on its current promotion of financial inclusion and microfinance in particular, stressing the need for significant sectoral reforms before this form of credit can be considered to align with the core principles of the DW programme. In particular, we would stress the need for further regulation with regards to loan amounts, interest rates, collateral considerations, flexibility in repayment schedules, and default mechanisms. In keeping with the promise to address ‘widespread vulnerability’ in Cambodia, and to ensure that ‘no one is left behind’, as stated in the latest Cambodia DW programme (ILO, 2019a), we would urge the ILO to take seriously demands from LICADHO and other campaigning organizations (CATU et al., 2020) to suspend the use of land titles as collateral. As highlighted here, the risk of losing homestead land acts as a severe form of labour discipline on precarious workers, compelling in some cases hyper-exploitative working conditions and even debt bondage. Finally, we would urge the ILO to interrogate the current structure of the Cambodian microfinance industry, with its commercial base, for-profit model and concurrent deregulation, and through this consider whether this sector and others similar to it in other countries can ever hope to engender ‘Decent Work’, given their divergent aims towards growth and shareholder returns. The links established in this article between microfinance on the one hand and not-decent-enough and indecent work on the other will also resonate in other country contexts with similarly large microfinance sectors, and highlevels of internal migration.

## Data Availability

Data from the Blood Bricks research project (ref: ES/R00238X/1) is held in the ReShare database, managed by UKRI. Data from the ‘ReFashion’ research project (ref: EP/V026054/1) will be available on the ReShare database once the project is complete. Data requests will be considered on an individual basis by Dr. Lawreniuk to access the data from her Leverhulme award.

## Footnotes

1 While the garment industry in Cambodia employs around a million workers, there exists a population of 6,863 brick workers nationally (Parsons and Ly Vouch 2019).

2 Interview with Vice President, trade union federation, 18.02.2020, Phnom Penh.

3 Interview with General Secretary, trade union federation, 19.02.2020, Phnom Penh.

4 Interview with Vice President, trade union federation, 18.02.2020, Phnom Penh, Interview conducted in English.

5 Interview with General Secretary, trade union federation, 18.02.2020, Phnom Penh.

6 Interview with General Secretary, trade union federation, 18.02.2020, Phnom Penh.

7 Interview with General Secretary, trade union federation, 19.02.2020, Phnom Penh.

8 Interview with brick worker, 03/11/2017; Phnom Penh.

9 Those indicated by workers were as follows: Sathapana, ACLEDA, PRASAC, Amret, AMK, Hatta Kakkasar and Vision Fund.

10 Interview with brick worker, 02.11.2017, Phnom Penh.

11 Interview with parents of brick worker, 16.11.2017, just outside Phnom Penh.

12 Despite the introduction of a maximum interest rate of 18% in 2017, our research on the ground after this policy was instigated suggests in a number of cases that microfinance institutions continued to levy higher interest rates, or introduce new charges to extract added returns outside of interest.

13 Interview in Svay Srok village, 28.01.2020.

## Code availability

n/a.

## References

[CR2] ADB. (2014). *Sector assessment: Agriculture, natural resources, and rural development, strategy and road map 2014–2018*. Manila: Asian Development Bank. https://www.adb.org/sites/default/files/linked-documents/cps-cam-2014-2018-ssa-01.pdf. Retrieved 16 June 2021.

[CR6] Arnold, D. (2021). *Cambodia’s garment sector in transformation external shocks, political space and supplier consolidation*. Phnom Penh: CNV International.

[CR7] Balkenhol, B. (1998). *Enterprise creation by the unemployed: The role of micro-finance; an ILO action program*. Geneva: Social Finance Unit, ILO. https://www.ciln.mcmaster.ca/papers/seconf/ilo.pdf. Retrieved 16 June 2021.

[CR8] Bargawi, O. (2005). *Cambodia’s garment industry: Origins and future prospects*. London: Overseas Development Institute. https://odi.org/en/publications/cambodias-garment-industry-origins-and-future-prospects/. Retrieved 16 June 2021.

[CR9] Bateman, M. (2017). The rise of Cambodia’s microcredit sector: An unfolding calamity (Conference paper). *Globalisation at the crossroads: Rethinking inequalities and boundaries, Bergen, August 2017.*

[CR204] Bateman, M., Natarajan, N., Brickell, K., & Parsons, L (2019). Descending into debt in Cambodia. *Made in China Journal*. https://madeinchinajournal.com/2019/04/18/descending-into-debt-in-cambodia.

[CR10] Bateman, M. (2020). *Microcredit in Cambodia: Why is there so much support for a failed poverty reduction model?* ISEAS—Yusof Ishak Institute. https://www.iseas.edu.sg/wp-content/uploads/2020/11/ISEAS_Perspective_2020_134.pdf. Retrieved 16 June 2021.

[CR11] Bateman M (2010). Why microfinance doesn’t work?.

[CR12] Bernards N (2016). The international labour organization and the ambivalent politics of financial inclusion in West Africa. New Political Economy.

[CR13] Breman J, van der Linden M (2014). Informalizing the economy: The return of the social question at a global level. Development and Change.

[CR208] Brickell, K., Parsons, L., Natarajan, N., and Chann, S. (2018). *Blood Bricks: Untold stories of modern slavery and climate change from Cambodia*. Royal Holloway, University of London. https://www.antislaverycommissioner.co.uk/media/1267/bloodbricks.pdf.

[CR14] Bylander, M. (2019). *Debt and the migration experience: Insights from South-East Asia*. International Organization for Migration report. Bangkok: IOM. https://thailand.iom.int/debt-and-migration-experience-insights-south-east-asia. Retrieved 16 June 2020.

[CR15] Bylander, M. (2020). Destination debts: Local and translocal loans in the migrant experience. *Geoforum*. 10.1016/j.geoforum.2020.06.015**(advance online publication)**.

[CR16] Bylander M (2014). Borrowing across borders: Migration and microcredit in rural Cambodia. Development and Change.

[CR17] Bylander M (2019). Is regular migration safer migration? Insights from Thailand. Journal on Migration and Human Security.

[CR18] Bylander M, Hamilton E (2015). Loans and leaving: Migration and the expansion of microcredit in Cambodia. Population Research and Policy Review.

[CR4] Cambodian Alliance of Trade Unions (CATU), Center for Alliance of Labor and Human Rights, and LICADHO. (2020). *Worked to debt: Over-indebtedness in Cambodia’s garment sector*. Joint Briefing Note. Phnom Penh: Cambodian Alliance of Trade Unions. https://www.licadho-cambodia.org/reports.php?perm=230. Retrieved 16 June 2021.

[CR20] Convergences. (2019). *Microfinance barometer 2019*. Paris: Convergences. http://www.convergences.org/en/104906-2/. Retrieved 16 June 2021.

[CR21] Cox R (1977). Labor and hegemony. International Organization.

[CR22] Crossa M, Ebner N (2020). Automotive global value chains in Mexico: A mirage of development?. Third World Quarterly.

[CR23] Dalal K, Aremu O, Ussatayeva G, Biswas A (2017). Out-of-pocket health expenditure and fairness in utilization of health care facilities in Cambodia in 2005 and 2010. F1000Research.

[CR24] Datta K, Martin R, Pollard J (2017). ‘Mainstreaming’ the ‘alternative’? The financialization of transnational migrant remittances. Handbook on the geographies of money and finance.

[CR25] Diepart JC, Ngin C, Oeur I (2019). Struggles for life: Smallholder farmers’ resistance and state land relations in contemporary Cambodia. Journal of Current Southeast Asian Affairs.

[CR26] Duvendack M, Mader P (2019). Impact of financial inclusion in low- and middle-income countries: A systematic review of reviews. Campbell Systematic Reviews.

[CR27] Frings V (1994). Cambodia after decollectivization (1989–1992). Journal of Contemporary Asia.

[CR29] Ghosh J (2013). Microfinance and the challenge of financial inclusion for development. Cambridge Journal of Economics.

[CR31] Gravesteijn, R. (2014) *Models of social enterprise? Microfinance organisations as promoters of decent work in Central Asia*. Bath: University of Bath. https://researchportal.bath.ac.uk/files/187957857/Thesis_with_copyright_material_removed.pdf. Retrieved 16 June 2021.

[CR32] Green, WN. (2020a). Financial landscapes of agrarian change in Cambodia. *Geoforum.*10.1016/j.geoforum.2020.02.001**(Advance online publication)**.

[CR33] Green WN (2019). From rice fields to financial assets: Valuing land for microfinance in Cambodia. Transactions of the Institute of British Geographers.

[CR34] Green WN (2020). Regulating over-indebtedness: Local state power in Cambodia’s microfinance market. Development and Change.

[CR35] Green WN, Estes J (2019). Precarious debt: Microfinance subjects and intergenerational dependency in Cambodia. Antipode.

[CR79] Greentech Knowledge Solutions [GKSPL]. (2016). *Introduction to Brick Kilns & specific energy consumption protocol for Brick Kilns*. New Delhi: GKSPL. https://www.ccacoalition.org/en/resources/introduction-brick-kilns-and-specific-energy-consumption-protocol-brick-kilns. Retrieved 16 June 2021.

[CR36] Guérin I (2014). The everyday use of microcredit in rural South India. Current Anthropology.

[CR200] Guermond, V. (2020). Contesting the financialisation of remittances: Repertoires of reluctance, refusal and dissent in Ghana and Senegal. *Environment and Planning A: Economy and Space*. 10.1177/0308518X20976141 (Advance online publication).

[CR201] Guermond V, Datta K, Mader P, Mertens D, van der Zwan N (2020). Financialization and/of migrant labor. The Routledge international handbook of financialization.

[CR37] Hauf F (2015). The paradoxes of decent work in context: A cultural political economy perspective. Global Labour Journal.

[CR38] Heibrink L (2019). The coercive power of debt: Migration and deportation of guatemalan indigenous youth. The Journal of Latin American and Caribbean Anthropology.

[CR39] Heng, D., Chea, S., & Heng, B. (2021) *Impact of interest rate cap on financial inclusion in Cambodia*. Washington DC: IMF. https://www.imf.org/en/Publications/WP/Issues/2021/04/29/Impacts-of-Interest-Rate-Cap-on-Financial-Inclusion-in-Cambodia-50349. Retrieved 16 June 2021.

[CR40] Hickey, S., & du Toit, A. (2007). *Adverse incorporation, social exclusion and chronic poverty*. 81. CPRC Working Paper. Manchester: Chronic Poverty Research Centre. https://www.chronicpoverty.org/uploads/publication_files/WP81_Hickey_duToit.pdf. Retrieved 16 June 2021.

[CR1] Human Rights Watch. (2015). *‘Work Faster or Get Out’: Labour rights abuses in Cambodia’s garment industry.* New York: Human Rights Watch. https://www.hrw.org/report/2015/03/11/work-faster-or-get-out/labor-rights-abuses-cambodias-garment-industry. Retrieved 16 June 2021.

[CR42] ILO. (2005). *Breaking the chains of poverty through microfinance.* Geneva: International Labour Organization*.* https://www.ilo.org/global/publications/world-of-work-magazine/articles/WCMS_081370/lang--en/index.htm. Retrieved 16 June 2021.

[CR43] ILO. (2007). *Microfinance for decent work: Organization and responsibilities of the social finance programme.* Geneva: International Labour Organization. https://www.ilo.org/wcmsp5/groups/public/---ed_emp/documents/publication/wcms_106329.pdf. Retrieved 16 June 2021.

[CR44] ILO. (2015). *Microfinance for decent work. Enhancing the impact of microfinance: Evidence from an action research programme*. Geneva: International Labour Organization. https://www.ilo.org/employment/Whatwedo/Publications/employment-reports/WCMS_344847/lang--en/index.htm. Retrieved 16 June 2021.

[CR47] ILO. (2019a). *Kingdom of Cambodia decent work country programme 2019–2023*. Phnom Penh: International Labour Organization. https://www.ilo.org/asia/publications/WCMS_710183/lang--en/index.htm. Retrieved 16 June 2021.

[CR48] ILO. (2019b). *Cambodia garment and footwear sector bulletin, Issue 9, July 2019*. Geneva: International Labour Organization. https://www.ilo.org/asia/publications/issue-briefs/WCMS_714915/lang--en/index.htm. Retrieved 16 June 2021.

[CR49] ILO (2019c). *Working on a warmer planet: The impact of heat stress on labour productivity and decent work*. Geneva: International Labour Organization. https://www.ilo.org/global/publications/books/WCMS_711919/lang--en/index.htm. Retrieved 16 June 2021.

[CR50] ILO (2021) *Decent work*. Geneva: International Labour Organization. https://www.ilo.org/global/topics/decent-work/lang--en/index.htm. Retrieved 16 June 2021.

[CR51] Johnson RL, Woodhouse M (2019). Securing the return: How enhanced US border enforcement fuels cycles of debt migration. Antipode.

[CR52] Kar S (2018). Financializing poverty: Labor and risk in Indian microfinance.

[CR53] Kunz Ra (2011). The political economy of global remittances: Gender.

[CR54] Kunz R, Maisenbacher J, Paudel LN (2020). The financialization of remittances: Governing through emotions. Review of International Political Economy.

[CR202] Lawreniuk, S., & Parsons, L. (2020). *Going nowhere fast: Mobile inequality in the age of translocality*. Oxford University Press.

[CR203] Lawreniuk, S. (2016). *Migration as distinction? Class, consumption and rural-urban migration in contemporary Cambodia*. PhD thesis: King's College London. https://kclpure.kcl.ac.uk/portal/files/57572302/2016_Lawreniuk_Sabina_1130286_ethesis.pdf.

[CR55] Lerche J (2012). Labour regulations and labour standards in India: Decent work?. Global Labour Journal.

[CR58] LICADHO. (2019) *Collateral damage: Land, loss and abuses in Cambodia’s microfinance sector*. Phnom Penh: LICADHO. https://www.licadho-cambodia.org/reports.php?perm=228. Retrieved 16 June 2021.

[CR59] Liv, D. (2013). *Study of the drivers of over-indebtedness of microfinance borrowers in Cambodia: An in-depth investigation of saturated areas*. Cambodia Development Resource Institute: Phnom Penh. https://sptf.info/images/oid-final-report.pdf. Retrieved 16 June 2021.

[CR60] Lundgren-Kownacki K, Kjellberg SM, Gooch P, Dabaieh M, Anandh L, Venugopal V (2018). Climate change-induced heat risks for migrant populations working at brick kilns in India: A transdisciplinary approach. International Journal of Biometeorology.

[CR61] Mader P (2015). The political economy of microfinance—financializing poverty.

[CR62] Mader P (2016). Card crusaders, cash infidels and the holy grails of digital financial inclusion. Behemoth - A Journal on Civilisation.

[CR63] Mader P (2018). Contesting financial inclusion. Development and Change.

[CR64] Mader P, Sabrow S (2019). All myth and ceremony? Examining the causes and logic of the mission shift in microfinance from microenterprise credit to financial inclusion. Forum for Social Economics.

[CR65] Mahanty, S., & Green, N. (2020) The evidence around Cambodia's microfinance debate. *Southeast Asia Globe.* https://southeastasiaglobe.com/unravelling-cambodias-microfinance-debate/. Retrieved 16 June 2021.

[CR66] Mosse D, Gupta S, Mehta M, Shah V, Rees J, FNMS, & KRIBP Project Team (2002). Brokered livelihoods: Debt, labour migration and development in tribal Western India. The Journal of Development Studies.

[CR205] Natarajan N, Brickell K, Parsons L (2019). Climate change adaptation and precarity across the rural–urban divide in Cambodia: Towards a ‘climate precarity’ approach. Environment and Planning E: Nature and Space.

[CR206] Natarajan N, Brickell K, Parsons L (2021). Diffuse drivers of modern slavery: From microfinance to unfree labour in Cambodia. Development and Change.

[CR68] Ovesen J, Trankell I (2014). Symbiosis of microcredit and private moneylending in Cambodia. The Asia Pacific Journal of Anthropology.

[CR207] Parsons, L. & Ly Vouch, L. (2019). A census of the Cambodian brick industry: Population, geography, practice. Brick Workers Trade Union of Cambodia.

[CR70] Rankin KN (2013). A critical geography of poverty finance. Third World Quarterly.

[CR71] Roberts A (2015). Gender, financial deepening and the production of embodied finance: Towards a critical feminist analysis. Global Society.

[CR72] Roy, A. (2010). *Poverty capital: Microfinance and the making of development*. Routledge.

[CR73] Saiag H (2020). Financialization from the margins. Focaal.

[CR74] Schicks J (2013). The sacrifices of micro-borrowers in Ghana – A customer-protection perspective on measuring over indebtedness.The. Journal of Development Studies.

[CR75] Schwittay A (2014). Making poverty into a financial problem: From global poverty lines to Kiva.org. Journal of International Development.

[CR76] Selwyn B (2019). Poverty chains and global capitalism. Competition & Change.

[CR77] Sinha, S. (2014) *Microfinance in Cambodia: Investors’ playground or force for financial inclusion?* European Microfinance Platform. http://www.e-mfp.eu/archive/201401. Retrieved 16 June 2021

[CR78] Soederberg S (2014). Debtfare states and the poverty industry: Money.

[CR81] Stoll, D. (2012). *El Norte or bust! How migration fever and microcredit produced a financial crash in a Latin American Town*. Rowman and Littlefield

[CR82] Taylor M (2012). The antinomies of ‘Financial Inclusion’: Debt, distress and the workings of Indian microfinance. Journal of Agrarian Change.

[CR83] Thoeun HC (2015). Observed and projected changes in temperature and rainfall in Cambodia. Weather and Climate Extremes.

[CR84] UN. (2015). *Sustainable development goals: 17 goals to transform our world.* United Nations. https://www.un.org/sustainabledevelopment/sustainable-development-goals/. Retrieved 15 June 2021.

[CR86] Vosko L (2002). `Decent Work’: The shifting role of the ILO and the struggle for global social justice. Global Social Policy.

[CR87] World Bank. (2018). *Riding the wave: An East Asian miracle for the 21st century.* World Bank, Washington DC. https://documents.worldbank.org/en/publication/documents-reports/documentdetail/770241511445721465/riding-the-wave-an-east-asian-miracle-for-the-21st-century. Retrieved 16 June 2021.

[CR89] Young S (2010). Gender, mobility and the financialisation of development. Geopolitics.

